# Genome-wide association study in Asia-adapted tropical maize reveals novel and explored genomic regions for sorghum downy mildew resistance

**DOI:** 10.1038/s41598-017-18690-3

**Published:** 2018-01-10

**Authors:** Zerka Rashid, Pradeep Kumar Singh, Hindu Vemuri, Pervez Haider Zaidi, Boddupalli Maruthi Prasanna, Sudha Krishnan Nair

**Affiliations:** 1 International Maize and Wheat Improvement Center (CIMMYT), ICRISAT Campus, Patancheru, Greater Hyderabad 502324 India; 2 International Maize and Wheat Improvement Center (CIMMYT), P. O. Box 1041, Village Market, Nairobi, 00621 Kenya

## Abstract

Globally, downy mildews are among the important foliar diseases of maize that cause significant yield losses. We conducted a genome-wide association study for sorghum downy mildew (SDM; *Peronosclerospora sorghi*) resistance in a panel of 368 inbred lines adapted to the Asian tropics. High density SNPs from Genotyping-by-sequencing were used in GWAS after controlling for population structure and kinship in the panel using a single locus mixed model. The study identified a set of 26 SNPs that were significantly associated with SDM resistance, with Bonferroni corrected P values ≤ 0.05. Among all the identified SNPs, the minor alleles were found to be favorable to SDM resistance in the mapping panel. Trend regression analysis with 16 independent genetic variants including 12 SNPs and four haplotype blocks identified SNP S2_6154311 on chromosome 2 with P value 2.61E-24 and contributing 26.7% of the phenotypic variation. Six of the SNPs/haplotypes were within the same chromosomal bins as the QTLs for SDM resistance mapped in previous studies. Apart from this, eight novel genomic regions for SDM resistance were identified in this study; they need further validation before being applied in the breeding pipeline. Ten SNPs identified in this study were co-located in reported mildew resistance genes.

## Introduction

Maize is the world’s leading cereal in terms of production, with 1,016 million metric tons (MMT) produced on 184 million hectares (M ha) globally^1^. The crop is produced in both temperate and tropical zones across all continents. Tropical maize is largely (about 80%) grown under rainfed conditions in sub-Saharan Africa, South and Southeast Asia, and Latin America, and is particularly vulnerable to an array of abiotic and biotic stresses^2^. In Asia, eight major maize growing countries (China, India, Indonesia, Nepal, Pakistan, Philippines, Thailand and Vietnam) together produce 98 per cent of Asia’s maize and 28 per cent of the world’s maize^3^. However, maize grown in the Asian tropics, especially during the monsoon season, is constrained an array of diseases, including downy mildews, leaf blights, rusts, stalk rots and ear rots. Among these diseases, the downy mildews (DMs) are widely prevalent across Asia, significantly affecting maize production and productivity. Globally, DM affected areas, including lowland tropical, subtropical, mid-altitude, transition zone and highland environments, suffer significant economic losses, reported to be as high as 30%^4^. Heavy losses (as high as 100%) due to different DM pathogens were reported on maize in several countries in Asia, including Indonesia, India, Nepal, Philippines and Thailand^5^. Disease transmission occurs mainly through oospores which survive in the soil^6^, but it can also be transmitted from plant to plant by airborne conidia or through infected seed. Because of the systemic nature of the disease, infected susceptible varieties usually die as the plants get infected at a very early stage, i.e., immediately after seedling emergence. When infection occurs during later stages of plant growth, the plants may survive, but without ear development. At least six pathogens that cause downy mildew are reported to infect maize in Asia, including sorghum downy mildew (SDM) caused by *Peronosclerospora sorghi* (Weston & Uppal).

Sorghum DM has a global distribution and is found at different altitudes and in different agro-ecological environments in the American, African, Australian and Asian subcontinents^7,8^. It causes considerable yield losses (as high as 30–40%) in several maize growing states of India, particularly Karnataka, Tamil Nadu and Rajasthan^9^. Despite the introduction of DM resistant cultivars in some countries and the extensive use of the systemic fungicide Metalaxyl, severe incidences of DM diseases are still reported in localized areas^10^. The pathogen’s resistance to Metalaxyl was reported by Raymundo^11^, and was also observed at Mandya, Karnataka, which is an SDM hot spot. Moreover, seed treatments with systemic fungicides make the seeds more expensive and beyond the reach of resource-poor farmers. Therefore, developing and deploying DM-resistant maize varieties is considered a cost-effective and environmentally-safer alternative for effectively overcoming the DM problem in maize.

Extensive studies on downy mildew resistance in maize were undertaken earlier through the CIMMYT co-ordinated Asian Maize Biotechnology Network (AMBIONET) in four Asian countries, namely India, Indonesia, Philippines and Thailand^12–14^. Limited variability was observed for resistance to some of the DMs, especially SDM^13,14^. Considering the quantitative nature of DM resistance, several groups undertook QTL mapping experiments in different countries with diverse mapping populations^5,15–19^. George *et al*.^5^ identified QTLs involved in resistance to all the major DM pathogens in Asia. Six genomic regions on chromosomes 1, 2, 6, 7 and 10 were identified for downy mildew resistance, including a major QTL on chromosome 6 that influenced resistance to five different DMs in the Asian region. Agrama *et al*.^15^ identified three QTLs for SDM resistance; two of them were closely mapped on chromosome 1 and the third one on chromosome 9. Sabry *et al*.^17^ detected three putative QTLs, one on chromosome 2 with large effect, and two on chromosomes 3 and 9 with minor effects. Studies by Jampatong *et al*.^18^ and Lohithaswa *et al*.^19^ identified QTLs for SDM resistance on chromosomes 2, 3, 4, 5, 6 and 9. However, the QTL mapping approach has several limitations, for example, it confines QTL to a 10–20 cM interval because of the limited number of recombination events during the development of the mapping population; also, only two alleles from each of the parents of the mapping population at any locus are studied in a biparental mapping population^20,21^.

A genome-wide association study (GWAS) is a powerful tool that identifies specific allele variants that confer improved resistance to various diseases in maize. It has been used in studying resistance to Fusarium ear rot^22^, gray leaf spot^23^, head smut^24^, northern corn leaf blight^25^, southern corn leaf blight^26^, sugarcane mosaic virus^27^, maize streak virus^28^ and maize lethal necrosis^29^. In Thailand, an association study on 60 inbred lines using 48 SSR markers identified the marker umc1033 as being significantly associated with SDM resistance^30^. As opposed to QTL mapping, which is a powerful method that identifies genomic regions and spans broad chromosome regions in a bi-allelic context, the main advantages of association mapping are increased mapping resolution, reduced research time, and the possibility of identifying a greater number of favorable alleles responsible for the trait of interest^31^.

CIMMYT has a long history of breeding for downy mildew resistance (DMR); as a result, specific populations like populations 22 (Mezcla Tropical Blanca), 28 (Amarillo Dentado), and 31 (Amarillo Cristalino-2) have been improved for DMR^32^. Four genetically broad-based populations were developed for DMR by the CIMMYT Asian Regional Maize Program (ARMP) and two sets of CIMMYT maize lines (CMLs) resistant to DM were released for use in DM resistance breeding in Asia. CIMMYT developed a panel of elite Asia-adapted tropical and subtropical maize inbred lines, called CIMMYT Asia Association Mapping (CAAM) panel. This panel enables association mapping studies for an array of major agronomic, biotic and abiotic stress tolerance traits in the region. The panel is particularly important for SDM analysis, considering the lack of adequate genetic variability for the trait in the region’s maize germplasm. DM resistant inbred lines developed in Thailand and Philippines as well as lines developed from DM resistant maize populations, such as early white DMR (EW-DMR), early yellow DMR (EY-DMR), late white DMR (LW-DMR) and late yellow DMR (LY-DMR) are included in the CAAM panel. Improved lines developed by the CIMMYT-Asia program by crossing elite materials tolerant to abiotic stresses like drought, heat and excess moisture with DMR lines were also included in the panel. Considering the CIMMYT ARMP’s history of incorporating resistance to DM, the CAAM panel is expected to have greater frequency of alleles favourable for DMR in the Asian region. The main objective of the present study was to identify genomic regions associated with SDM resistance and to validate previously reported QTL responsible for DM resistance in maize.

## Results

### Phenotypic evaluation for SDM resistance

The CAAM panel with a set of 368 lines was evaluated against SDM at Mandya, India, during the years 2012 and 2015 under artificial epiphytotic conditions. The disease severity was high in both years, with the standard susceptible check CM500 showing 100 per cent mortality. The mean disease severity of the trials was also high, with 96.28 and 89.21 per cent during 2012 and 2015, respectively. A Box-Cox transformation lambda (λ) value of 0.96 was obtained and used to transform the data. There was significant genotypic variance (p ≤ 0.001) for the trait in the panel and the broad-sense heritability estimated by the combined analysis was high (0.72). Heritability estimates for 2012 and 2015 were 0.92 and 0.74, respectively (Table 1).

**Table 1:** Estimates of mean, variance components and heritability of SDM responses in the CAAM panel evaluated at Mandya, Karnataka, during the years 2012 and 2015.

Year	Mean	Min	Max	CV	Genotypic variance	G x Y variance	Error variance	Heritability
2012	96.28	1.45	100	5.05	464.16**		73.43	0.92
2015	89.21	0	100	9.58	109.08**		73.43	0.74
Across	92.75	5.32	100		215.04	142.19	48.64	0.72

**P value ≤ 0.001.

### Population structure and linkage disequilibrium (LD) decay

**Figure 1:**
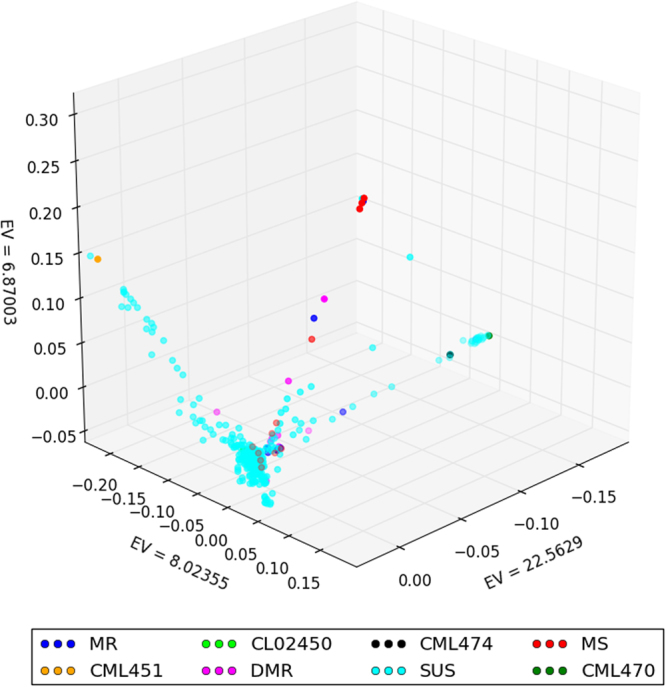
Population structure based on the first three eigenvalues of principal component (PC) analysis of the CAAM panel using 63,546 SNPs. Clusters in dark blue represent moderate resistant (MR) lines, florescent green (CL02450) and black (CML474) dots represent tester lines of CIMMYT’s heterotic group A, red dots are moderately susceptible (MS) lines, pink are downy mildew resistant (DMR) lines, and clusters of florescent blue dots are susceptible lines (SUS), while yellow (CML451) and green (CML470) dots represent CIMMYT’s heterotic group B tester lines.

Principal component analysis using 63,546 SNPs (CR 0.9, and MAF ≥0.1 with LD pruning) did not reveal a clear population structure with the first three principal components (Fig. 1). All the important tester lines from various maturity groups used in the Asian tropics and mapped within the 3D PCA plot revealed the spread of SDM resistant lines within the studied panel. Two early maturing testers developed in the Asian tropics, CML470 and CML474, clearly grouped into one cluster in the PCA plot. The Scree plot plotted (Supp. Figure 1) with all the eigen values suggested 10 principal components that should be considered in the GWAS to account for the population structure.

**Figure 2:**
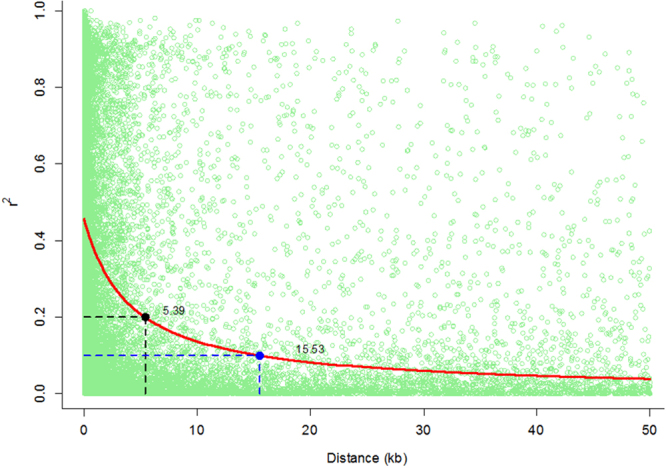
Linkage disequilibrium (LD) plot representing the average genome-wide LD decay of the CAAM panel using 37,043 genome-wide SNP markers. The values on the Y-axis represent the squared correlation coefficient r^2^ and the X-axis represents the genetic distance in kilobases (Kb).

Genome-wide LD across 37,043 SNPs with minor allele frequency (MAF) ≥0.3 was investigated in the CAAM panel. The genome-wide LD decay was plotted as LD (r^2^) between adjacent pairs of markers versus distance in kb which showed that LD decay was 15.53 Kb at r^2^ = 0.1 and 5.39 kb at r^2^ = 0.2, (Fig. 2). Chromosome-wise LD analysis showed fastest LD decay in chromosome 5 (10.35 Kb at r^2^ = 0.1 and 3.59 Kb at r^2^ = 0.2), followed by chromosome 2 (11.39 Kb at r^2^ = 0.1 and 3.95 Kb at r^2^ = 0.2). However, the slowest LD decay was observed on chromosome 8 (28.8 Kb at r^2^ = 0.1 and 9.99 Kb at r^2^ = 0.2), followed by chromosome 4 (22.14 Kb at r^2^ = 0.1 and 7.68 Kb at r^2^ = 0.2) in this panel (Supp. Table 1).

### Marker-trait associations for SDM resistance

**Figure 3:**
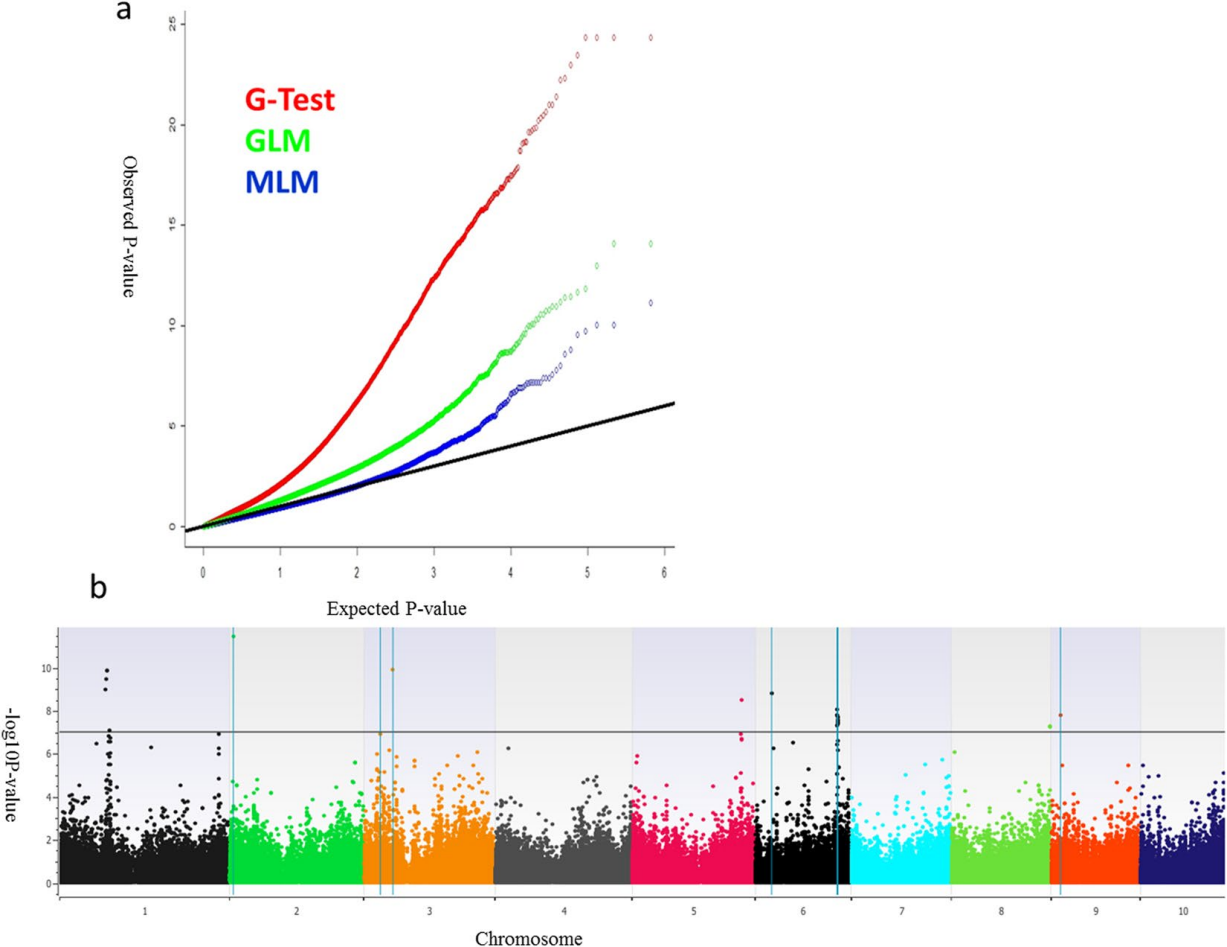
(**a**) Inflation depicted by Q-Q plots of observed versus expected −log_10_ (P values) plots for SDM using the naïve association model (G-test), GLM (G + Q) and MLM (G + Q + K); G = genotype (fixed), Q = ten principal components (fixed), K = kinship matrix (random); (**b**) Highly significant SNPs identified from MLM model using Manhattan plot, plotted with the individual SNPs on the X-axis and −log10 *P* value of each SNP on the Y-axis. The blue vertical lines show the significant associations at previously reported QTLs.

A subset of markers with a call rate ≥0.7 and minor allele frequency ≥0.03 were used for association studies. With the total 333,039 SNPs fulfilling these criteria, a naïve association analysis with only these SNPs was carried out. The Quantile-quantile plot (QQ plot) drawn using the observed against the expected -log_10_ (P values) showed significant genomic inflation (Fig. 3a). Following this, to control the genomic inflation, a general linear model analysis including the genotypes and 10 PCs (G + Q) was carried out, which also showed high genomic inflation in the QQ plot. Further, a single marker mixed linear model including the genotypes, 10 PCs and the kinship matrix (G + Q + K) was able to keep the genomic inflation within acceptable limits. Based on this analysis, 26 significant SNP hits for disease response were identified that had Bonferroni-corrected P values ≤ 0.05 on chromosomes 1, 2, 3, 5, 6, 8 and 9 (Fig. 3b; Supp. Table 2). SNP S2_6154311 on chromosome 2 showed the strongest association with the lowest P value (1.0E-012) and Bonferroni-corrected P value (1.0E-06). The phenotypic variance explained by significant SNPs ranged from 5.50 to 13.07%; SNP S2_6154311 and S1_88004654 explained the highest and lowest proportion of variances, respectively, from among the SNPs associated with DM resistance (Supp. Table 2). Interestingly, in all 26 associations identified, the minor allele was found to contribute positively towards lowering SDM incidence (Supp. Table 2). Lines developed by crossing DM resistant lines with elite inbred lines (including CMLs) developed from the DM resistant populations were found to carry the maximum number of favourable alleles in the CAAM panel. A number of these SNPs were within the same chromosomal bins as the QTLs previously mapped for SDM resistance in multiple studies (Table 2). Annotated genes in the reference genome of B73 RefGen_V2 (available at http://ensembl.gramene.org/Zea_mays) carrying the 26 associated SNPs showed many genes reported to be associated with broad spectrum disease resistance or resistance specifically to mildews (Table 3).

**Table 2:** SNPs identified in GWAS compared with previously reported QTL on chromosome bins of the maize genome for downy mildew resistance.

QTL interval	Chr. bin	Population studied	GWAS-based SNPs	References
umc2363-umc1165	2.02	F_2:3_	S2_6154311	^19^
umc1223-bnlg420	3.04–3.05	BC_1_F_1_	S3_28841309 S3_51326624	^16^
umc2002	3.04	F_2:3_	S3_28841309 S3_51326624	^18^
bnlg1867	6.01	F_2:3_	S6_28477908	^18^
phi077-bnlg107	6.01	F_2:3_	S6_28477908	^19^
bnl8.23-bnl5.47a	6.05	RILs	S6_145310940 S6_145310942 S6_145312028 S6_145321547 S6_145432233 S6_145432240 S6_145491591	^5^
mmc0241-umc1859	6.05	BC_1_F_1_	S6_145310940 S6_145310942 S6_145312028 S6_145321547 S6_145432233 S6_145432240 S6_145491591 S6_146125265	^16^
umc1033	9.02	Inbred Lines	S9_16183526	^31^

**Table 3:** Significant SNPs associated with SDM resistance within the predicted candidate genes and their reported functions in crop plants.

Marker	Gene	Active domain	Crop	Disease	References
S1_81579624	GRMZM2G178079	Glutathione S-transferase	Maize	Multiple disease resistance	^69^
S1_82633548	—	—	—	—	—
S1_84567152, S1_84567165	GRMZM2G040441	MLO-like protein	wild grass (Cereal model Species) Brachypodium distachyon, barley, tomato, arabidopsis, maize	Powdery mildew	^42,43^
S1_88996380	GRMZM2G089248	Exostosin, Glycosyltransferase Family 47	Arabidopsis	Diseases caused by Fusarium	^70,71^
S1_283192413	GRMZM5G867796	—			—
S2_6154311	GRMZM5G824964	EB1 C-terminal	—	—	^72–74^
S3_28841309	GRMZM2G104088	Transmembrane & Transmembrane helix	Arabidopsis	Bacterial speck, downy mildew	^75^
S3_51326624	GRMZM2G000107	Peroxidase	Maize, rice	Maize dwarf mosaic virus, bacterial blight	^76,77^
S5_193536588	GRMZM2G465957	Protein kinase	Maize, rice	Southern corn leaf blight, bacterial blight	^78,79^
S5_193651730	GRMZM2G005996	Transmembrane, Transmembrane helix	Arabidopsis	Bacterial speck, downy mildew	^75^
S6_28477908	GRMZM2G124297	HECT (E6AP-type E3 ubiquitin-protein ligase)	Arabidopsis	Downy mildew; plant innate immunity	^50–52^
S6_145310940, S6_145310942, S6_145312028	GRMZM2G479523	Cupin_1	Barley, wheat	Powdery mildew	^45,46^
S6_145321547	GRMZM2G479608	Transmembrane BAX inhibitor motif-containing protein	Barley, arabidopsis	Powdery mildew, programmed cell death	^48,49^
S6_145432233, S6_145432240, S6_145491591	GRMZM2G108057	Cation-transporting ATPase	Tomato, barley	Leaf mold, powdery mildew	^47^
S6_146125265	GRMZM2G173630	Abhydrolase_3	Lemon plant	*Citrus huanglongbin*	^80^
S8_173851044, S8_173851047, S8_173851049, S8_173851050, S8_173851053	GRMZM2G031724	Gibberellin 2- beta oxidase	—	—	—
S9_16183526	GRMZM2G104760	Protein kinase	Maize, rice	Southern leaf blight, bacterial blight	^78,79^

(—)Information not available.

### Haplotype estimation and trend regression analysis

The 26 SNPs identified as being significantly associated with SDM incidence were spread across seven chromosomes. Some of these SNPs had physical coordinates (based on B73 RefGen_V2) that were in close proximity, and some of them were SNPs located inside the same annotated genes. Hence haplotype block estimation analysis was carried out to determine whether these SNPs form haplotype blocks using the haplotype defining algorithm to minimize historical recombinations. In this analysis, four haplotype blocks were detected on three chromosomes from among the 26 SNPs associated with SDM incidence. Based on this analysis, 16 independent variants (including both SNPs and haplotype blocks) were considered for further analysis. Trend regression analysis on these 16 variants against SDM incidence estimated the proportion of variance explained by each of these to range from 3.61 to 26.74% (Table 4). S2_6154311 contributed 26.74% of the variation, followed by Hap-1, which contributed 17.34% of the variation.

**Table 4:** Trend regression analysis using significant SNPs and haplotype blocks for SDM resistance in the CAAM panel.

Haplotype blocks/ SNPs	Chr.	Markers used	Alleles/ Haplotypes	P-Value	R Squared	Bonferroni P	Favourable allele	Novel/previously reported
SNP-1	1	S1_81579624	2	0.000388	0.036	0.0062126	G	Novel
SNP-2	1	S1_82633548	2	0.000424	0.0361	0.0067875	A	Novel
Hap_1	1	S1_84567152, S1_84567165	2	2.85E-11	0.173	4.56E-10	AT	Novel
SNP-3	1	S1_88996380	2	4.70E-07	0.08	7.53E-06	G	Novel
SNP-4	1	S1_283192413	2	5.26E-05	0.0478	0.0008424	C	Novel
SNP-5	2	S2_6154311	2	2.61E-24	0.267	4.18E-23	G	previously reported^19^
SNP-6	3	S3_28841309	2	1.45E-08	0.092	2.32E-07	A	previously reported^18^
SNP-7	3	S3_51326624	2	1.28E-12	0.137	2.05E-11	C	previously reported^18^
SNP-8	5	S5_193536588	2	9.82E-10	0.105	1.57E-08	G	Novel
SNP-9	5	S5_193651730	2	3.36E-05	0.049	0.0005381	A	Novel
SNP-10	6	S6_28477908	2	3.57E-06	0.077	5.71E-05	T	previously reported^18^
Hap_6.1	6	S6_145310940, S6_145310942, S6_145312028, S6_145321547	3	1.07E-08	0.124	1.71E-07	ACCC	previously reported^5,16^
Hap_6.2	6	S6_145432233, S6_145432240, S6_145491591	2	3.01E-08	0.106	4.81E-07	TCG	previously reported^5,16^
SNP-11	6	S6_146125265	2	1.39E-06	0.073	2.22E-05	T	previously reported^5,16^
Hap_8	8	S8_173851044, S8_173851047, S8_173851049, S8_173851050, S8_173851053	2	3.79E-11	0.16	6.06E-10	AGGGG	Novel
SNP-12	9	S9_16183526	2	2.87E-06	0.064	4.59E-05	A	previously reported^30^

## Discussion

The downy mildews of maize caused by *Peronosclerospora* species are a group of economically important and widespread diseases in many tropical and subtropical regions of the world^5^. The lack of adequate and genetically diverse sources of resistance to DMs has been a major constraint in tropical Asia, especially in the maize growing environments of South and Southeast Asia. Introgressing DM resistance in the breeding pipeline is one of the priorities of CIMMYT’s maize breeding programs as well as National Agricultural Research System (NARS) partners in Asia. Breeding for DM resistance, especially phenotyping under artificial epiphytotic conditions with high disease pressure is cost-intensive. Therefore, identifying, validating and deploying molecular markers with DM resistance can increase the efficiency of developing DM resistant tropical and subtropical maize germplasm especially in those countries where DM resistance is mandatory for the release of maize varieties.

The CAAM panel used in this study includes tropical/subtropical inbred lines from CIMMYT breeding programs in Asia, Mexico, Kenya, Zimbabwe and Colombia, that are also adapted to the Asian tropics. Derivatives of several lines developed by crossing CIMMYT populations with Suwan sources of DMR from Thailand, and the Asian mildew acid tolerant late (AMATL) populations are included in the CAAM panel. This panel is an excellent resource to study genomic regions for SDM resistance considering CIMMYT ARMP’s breeding efforts aimed at improving DM resistance of Asian maize germplasm. Heritability estimates of the trials evaluated at Mandya were high (0.74–0.92); for the two years (2012 and 2015) combined, the SDM scores ranged from 7.81 to 100%, indicating there is genetic variability for SDM in the CAAM panel. Genotypic variance was also highly significant for SDM resistance in the CAAM panel. Lines showing the lowest disease percentage were CML433, (CML474/S92145–2EV-7-3-B*5)-F2-25-1-B, CML472, CA14709-4-7-5-1, which were either developed for resistance to DM for the Asian region or derived from crosses with DMR lines.

A GBS protocol commonly used by the maize research community was applied in this study, which produced a partially imputed SNP dataset, calling about a million SNPs. For principal component and kinship analysis, high quality SNPs with filtering criteria of CR ≥0.9 and MAF ≥0.1, and pruned at an r^2^ threshold of 0.5 were used. It was necessary to reduce the number of SNPs for computational efficiency, but by selecting SNPs that have higher MAF and by LD-pruning, due care was taken to avoid confounding of the calculations due to large blocks of SNPs that have strong LD with each other^33^. The PCA showed only a moderate population structure within the CAAM panel, with no clear grouping of the lines into well-defined clusters. The first three PCs explained only 39.3% of the variation in the panel. The DMR lines did not fall into a particular cluster, but were scattered among the groups. This was also observed by George *et al*.^14^ who studied the diversity pattern of a group of Asian inbred lines along with CIMMYT’s tropical and subtropical lines, and suggested that the tropical and subtropical lines in the Asian region possess significant genetic diversity that did not allow a clear-cut distinction into well-defined clusters. Warburton *et al*.^34^ observed that the CIMMYT populations that served as germplasm sources for many Asian lines had a large amount of diversity within, rather than between, source populations. Due to this heterogeneous nature of the CIMMYT populations, they suggested that it would be difficult to find a well-defined population structure in the Asian maize lines. Genetic diversity is directly related to LD, which in turn affects LD based association mapping; the larger the LD blocks, the slower the LD decay, which will result in lower mapping resolution^35,36^. For estimating genome-wide LD, we used a subset of the SNP data filtered for CR ≥0.9 and MAF ≥0.3. This was also done for computational efficiency required for the analysis, with due care taken for SNP selection to represent all population structure groups by way of increasing MAF. The same set of SNPs used for PCA were not used in this analysis as the former set had already been pruned at an r^2^ threshold of 0.5. In the CAAM panel, the average LD decay of the panel was 15.53 kb at r^2^ = 0.1 and 5.39 kb at r^2^ = 0.2, with localized differences within and between chromosomes. The slowest LD decay was observed in chromosome 8, and a similar observation was made by Suwarno *et al*.^35^, while studying a tropical/subtropical carotenoid association mapping panel. LD decay in tropical maize germplasm is generally found to be high as compared to temperate maize germplasm. Lu *et al*.^36^ suggested the LD decay distance in temperate maize germplasm (10 to 100 kb) was 2 to 10 times higher than that of tropical maize germplasm (5 to 10 kb). Similarly, Romay *et al*.^37^ studied 2,815 inbred lines and found LD decay of 1 kb for tropical maize germplasm and 10 kb for temperate germplasm. The high LD decay of tropical maize germplasm suggested that it has a broader genetic base resulting from high recombination events and might have more rare alleles than temperate lines^38^; this provides an excellent opportunity for maize breeders to select germplasm that combines higher grain yield with disease resistance and relevant abiotic stress tolerance traits.

QTL mapping for DM resistance was reported by many groups on multiple DMs in diverse germplasm and environments^5,15–19,31^. A genome-wide study using high density marker information from widely adapted maize lines has not yet been reported. On Asia-adapted CIMMYT maize lines genotyped at high density, three methods of association testing were employed in this study; the single locus mixed linear model was eventually selected for reporting results, as it was successful in controlling genomic inflation by correcting for population structure and familial relatedness^39^. A set of 26 SNPs on seven chromosomes were found to be significantly associated with SDM resistance (Supp. Table 2). Since many of the SNPs identified were having strong LD in average pairwise LD analysis (Sup. Table 3), we carried out haplotype block detection among those SNPs. We estimated the haplotype frequencies and identified 16 genomic regions in linkage equilibrium, either as SNPs or haplotype blocks. The SNP alleles/haplotypes were regressed against the phenotype to ascertain the genomic regions that contributed most significantly to DM resistance. SNP/haplotype-trait associations identified were assessed by way of previously reported QTL in these genomic regions, and functional domains in the predicted gene models carrying the SNPs. This will further be followed up by validation in independent bi-parental populations, before deployment in breeding pipeline.

The most significant association was found to be on chromosome 2 (SNP S2_6154311; Bonferroni-corrected p value = 3.92E-23) contributing 26.70% to phenotypic variation. The SNP S2_6154311 was found to be located in bin 2.02 according to the Maize Genome Data Base (WWW.maizegdb.org, Maize B73 RefGen_V2). Lohithaswa *et al*.^19^ identified a QTL for SDM resistance in the chromosomal bin 2.02 between SSR markers umc2363 and umc1165 (Table 3). Nair *et al*.^16^ identified QTL on chromosome 3, bin 3.04/3.05 (between SSR markers umc1223 and bnlg420) in a backcross mapping population that was also phenotyped at Mandya for SDM resistance. Jampatong *et al*.^18^ also identified a QTL on chromosome 3.04 for SDM resistance in their study on an F_2:3_ mapping population. Our study also identified two highly significant (Bonferroni value ≤ 0.05) SNPs, S3_28841309 and S3_51326624, on chromosome 3 (bin 3.04) for SDM resistance, explaining 9.20 and 13.80% of the phenotypic variance, respectively. This particular chromosomal bin was found to be harbouring clusters of disease resistance genes^40^ for as many as six of the 11 diseases in a meta-analysis study^41^. Phumichai *et al*.^30^ identified significant SSR marker umc1033 linked with DM resistance on chromosome 9 (bin 9.02) in the association studies. In our study, SNP S9_16183526 (Bonferroni value ≤ 0.05) located on chromosome 9 in bin 9.02 was identified for DM resistance.

Another most important genomic region for DMR is on chromosome 6, which has been identified in many studies^5,16,18^. In our study, nine SNPs were found to be significantly associated with SDM resistance on chromosome 6, and seven of them formed two haplotype blocks. One SNP and two haplotype blocks were located on bin 6.05, where a significant QTL was identified by George *et al*.^5^ for multiple DM resistance against five different pathogens including the SDM pathogen. Nair *et al*.^16^ also identified a major QTL on chromosome 6.05 for SDM and RDM in their backcross population. The significant associations identified in this region in our GWAS add strength to the earlier findings, and could be taken up for further analysis and potential application in maize breeding programs. Another SNP identified on chromosome 6, SNP S6_28477908, is located in bin 6.01; two earlier studies identified SDM resistance QTL in this bin^18,19^. Chromosome 6 is also reported to harbour clusters of resistance genes for multiple biotic stresses, and bin 6.01 was reported to have an array of resistance genes, as in the case of chromosomal bin 3.04^40^. The other significant associations identified in this study seem to be novel, and were not found in the previous QTL mapping studies on SDM resistance (Table 4).

### Candidate genes associated with SDM

Generally, plant disease resistance is achieved through various genes acting on mechanisms ranging from innate plant immunity to broad spectrum basal defence mechanisms to specialized mechanisms targeting specific pathogens. Many of the SNPs that were found to be associated with SDM resistance in this study were located in annotated genes (B73 RefGen_V2) with functional domains that were previously reported to influence disease resistance in various crops (Table 3). Mildews, including both downy mildews and powdery mildews, are caused by obligate biotrophic pathogens that require a live host for their survival. At least 10 out of the 26 SNPs identified as significant in the present study were localized within five different genes that had been previously implicated in mildew resistance in different crops. A novel haplotype identified on chromosome 1 was located within GRMZM2G040441, which has an active MLO-like domain. An MLO protein was cloned initially from barley as the resistance gene against powdery mildew^42,43^, and thereafter, many orthologs and paralogs for the MLO gene were identified and characterized in great detail in many plants^44^. MLO proteins are reported to have characteristic domain features which aid the defence signalling mechanism. Though we have not encountered reports on MLO-like proteins involved in downy mildew resistance, it would be worthwhile to investigate the role of MLO-like proteins in SDM resistance in maize.

Significantly associated SNPs on chromosome 6 were located in four genes, three of which are adjacent genes predicted on bin 6.05. Whether the association of many SNPs in close proximity is due to high LD in the region or whether there is a cluster of genes operating towards a resistance reaction needs further investigation. Three significant associations on chromosome 6 were discovered within the Cupin super family gene. Germins, which are part of this super family, have been reported to be involved in powdery mildew resistance in wheat and barley^45,46^. Similarly, three significantly associated SNPs were found be located within GRMZM2G108057, putatively coding for a cation transporting ATPase. Regulation of cation ATPase is involved in one of the earliest cellular responses during activation of plant immune responses by modulation of extracellular pH and acidification of the apoplast, which was observed in incompatible interactions of barley plants with the powdery mildew fungus *Blumeria graminis* ^47^. Another closely located gene, GRMZM2G479608, is predicted to code for a transmembrane Bax inhibitor motif containing protein 4. In barley, over-expression of the Bax Inhibitor 1 gene was found to breakdown mlo mediated penetration resistance against powdery mildew^48^. Watanabe *et al*.^49^ concluded that Bax Inhibitor 1 was a conserved plant cell death suppressor, which is a key molecular switch downstream from a range of stress signals. On chromosome bin 6.01, a significant association was detected with a SNP located in gene GRMZM2G124297 predicted to code for the HECT sub-class of E3 Ubiquitin ligases. This class of proteins have been reported to provide innate immunity in plants^50^. E3 Ubiquitin ligases were also found to be involved in SAR independent resistance to the downy mildew pathogen in *Arabidopsis thaliana* ^51,52^. As many as eight novel associations were identified in this study, and five of them were within predicted genes with functional domains implicated in disease resistance. Though the correlations identified in association studies cannot be dubbed as causations, associated SNPs on genes previously reported to be trait related add much value and strength to the results to carry forward with independent validation in biparental populations and functional studies of the candidate genes.

## Conclusion

The present study is the first one to report significant SNPs and haplotypes associated with SDM resistance in maize, based on GWAS. Six of these SNPs/haplotypes are located within major QTL intervals reported in previous mapping studies on biparental populations, and hence, could be of value to maize breeding programs in tropical Asia that are intensively engaged in developing and deploying DM resistant elite maize varieties. Also, since ten marker-trait associations were identified within candidate genes implicated in disease resistance, especially mildew resistance, the findings from the present study could serve as a strong base for future functional studies to dissect resistance to downy mildews in maize.

## Methods

### Germplasm

The CAAM panel consisted of 419 inbred lines that were developed and adapted in Asia, based on a global collection of maize germplasm available from CIMMYT. It involved inbred lines with tolerance to abiotic stresses like drought, high temperature and excess moisture, besides Quality Protein Maize (QPM) lines, and lines developed from downy mildew resistant populations in Asia. The CAAM panel includes lines that are adapted to tropical, subtropical, lowland, mid-altitude and highland environments, and were classified as early maturing, intermediate maturing and late maturing based on growing degree days (GDD).

### Phenotypic evaluation

The CAAM panel, with a set of 368 lines, was phenotyped for SDM (*Peronosclerospora sorghi*) at the hot-spot location of Mandya, Karnataka, India (12°N; 76°E; 695 masl; 705 mm/year average annual rainfall) during the monsoon season in 2012 and 2015. Mandya was also identified as the location with the highest DM susceptibility scores, along with Maros in Indonesia, in an earlier study that evaluated a common mapping population across five locations in Asia^5^. Artificial epiphytotic conditions were created following the infector row method by planting ‘infector rows’ of CM500, 30 days before planting the test entries (in the first week of July). The infector rows were planted on all sides of the experimental block, and also on one bed (2 m wide) after every two beds of test entries (each 3 m wide) to ensure uniform disease pressure on the test entries across the field. Fresh conidia were collected early in the morning (between 2 and 3 am) from 3- to 4-week-old systematically infected maize plants in the DM nursery by suspending the severely infected leaves in water. The conidia suspension was sprayed on seedlings (2–3 leaf stage) in the infector rows using hand-sprayers immediately after collection. The inoculation process was repeated continuously for 7–10 days to achieve good disease incidence on infector rows. Test entries were planted in one-row plots about 30 days after infector row planting (in the first week of August, when relative humidity was >90%), at the time when DM infection on infector row plants reached >70%. Each test entry was planted in standard 3-m-long rows with plant-to-plant spacing of 20 cm and row-to-row spacing of 75 cm in an alpha-lattice design with two replications. Similar to the infector rows, conidial suspension was collected from the DM nursery and sprayed on the test entries and disease indicator rows, starting from 7 to 8 days after seedling emergence, and during the following 7 days to avoid any chance of escape^53^. Severe SDM incidence (>95%) on all indicator rows of susceptible check entries planted along with the test entries was considered an indication of uniform and sufficient disease pressure throughout the trial.

### Disease scoring

Percentage disease incidence was calculated as the number of SDM infected plants at 21 and 35 days after emergence compared to the total number of plants per plot. Disease percentage at 35 days was considered as the final score because the disease score remained constant after 35 days. A DM rating was recorded using a modified rating method suggested for maize^54^, i.e. 0% = highly resistant, 1–10% = resistant, 11–25% = moderately resistant, 26–50% = moderately susceptible, 51–75% = susceptible, and 76–100% = highly susceptible.

### Phenotypic data analysis

The phenotypic data on percentage disease scores were skewed towards susceptibility in the CAAM panel. The data were subjected to Box-Cox power transformations^55^, which helped the data approach normality. Best Linear Unbiased Estimators (BLUEs) obtained using the software METAR-4.1^56^ from multi-location data analysis were used for GWAS analysis. Analysis of variance components were estimated using Genstat (14^th^ edition)^57^. Broad-sense heritability of the combined analysis across years was calculated as H^2^ = σ^2^_g_/(σ^2^_g_ + σ^2^_ge_/*e* + σ^2^_e_/*er*), where σ^2^_g_, σ^2^_ge_ and σ^2^_e_ are the genotypic, genotype-by-year interaction and error variance components, respectively, and e and r are the number of years and number of replicates within each year included in the corresponding analysis, respectively.

### DNA isolation and genotyping

DNA of all inbred lines was isolated from the leaf samples of 3–4-week-old seedlings using the standard CIMMYT laboratory protocol^58^. Genotyping was carried out on the GBS platform^59^ at the Institute of Genomic Diversity, Cornell University, Ithaca, USA. Genomic DNA was digested with the restriction enzyme ApeK1. GBS libraries were constructed in 96-plex and sequenced on Illumina HiSeq. 2000. SNP calling was performed using the TASSEL-GBS pipeline with B73 as the reference genome^60^ to generate a comprehensive genotype collection, the AllZeaGBSv2.7 Production Build (www.panzea.org). The TASSEL-GBS pipeline is optimized for low sequencing depth (0.5 to 3×) over a large number of markers in a large sample of individuals. This collection included genotypes of more than 60,000 maize samples. In this study, we focused on the subset of 368 lines forming the CAAM panel, and partially imputed raw GBS data were used for further genetic analyses. The original partially imputed data set consisted of 955,690 SNPs across all the chromosomes, which included partially imputed data based on an algorithm that searched for the closest neighbour in small SNP windows across the entire maize database, allowing for a 5% mismatch^37^. If the requirements were not met, the SNP was not imputed, leaving only about 10% of the data unimputed. For GWAS, filtration criteria of call rate (CR) ≥0.7 and minor allele frequency (MAF) ≥0.03 were used, yielding 333,039 SNPs. For calculating PCA and kinship matrix, high quality SNPs with filtering criteria of CR ≥0.9 and MAF ≥0.1, and pruned at an r^2^ threshold of ≤0.5 were used for selecting 63,546 SNPs. Filtering criteria of CR ≥0.9 and MAF ≥0.3 generated 37,043 SNPs for the LD adjacent pair analysis used for calculating r^2^.

### Principal component and kinship analysis

The PCA method described by Price *et al*.^61^ was implemented in the SNP & Variation Suite (SVS) V_8.6.0 (SVS, Golden Helix, Inc., Bozeman, MT, www. goldenhelix.com). A three-dimensional plot of the first three principal components was drawn to visualize the possible population stratification among the samples. A scree plot was plotted to determine the number of principal components to be included in the GWAS analysis. A kinship matrix was computed from identity-by-state distances matrix as executed in SVS V_8.6.0., where IBS distance = (no. of markers IBS2) + 0.5 × (no. of markers IBS1) no. non-missing markers, where IBS1 and IBS2 are the states in which the two inbred lines share one or two alleles, respectively at a marker^62^.

### LD analysis and haplotype trend regression

The extent of genome-wide and chromosome-wise linkage disequilibrium (LD) was based on adjacent pairwise r^2^ values (the squared correlation coefficients among alleles at two adjacent SNP markers) between adjacent SNPs among 37,043 high quality SNPs from the GBS data and physical distances between those SNPs^63^. Nonlinear models with r^2^ as responses (y) and pairwise distances (x) as predictors were fitted into the genome-wide and chromosome-wise LD data using the ‘nlin’ function in R^64^. Average pairwise distances in which LD decayed at r^2^ = 0.2 and r^2^ = 0.1 were then calculated based on the model given by Hill & Weir^65^.

LD analysis and haplotype frequency estimation were also done among all the SNPs found to be associated with SDM resistance using the Expectation Maximisation (EM) algorithm^66^ as implemented in SVS V_8.6.0. Haplotype frequencies were estimated from among the SNPs associated with the trait using 50 EM iterations, an EM convergence tolerance of 0.0001 and a frequency threshold of 0.01. Haplotype blocks were detected based on the block-defining algorithm to minimise historical recombinations^67^. Trend regression analysis of the haplotypes and SNPs was carried out based on a stepwise regression of the SDM phenotype with the pre-estimated haplotypes with forward elimination and a P-value cut-off of 0.001.

### GWAS

GWAS was carried out on the SDM phenotype based on three methods: using only genotype data (G; Naïve model), using genotype data corrected for population structure (G + Q; General linear model (GLM)) and using genotype data corrected for both population structure and kinship (G + Q + K; single locus mixed linear model (MLM)). These analyses used genotype association tests with an additive model for the first two analyses and a mixed model using single locus (EMMAX)^68^ for the third analysis as implemented in SVS V_8.6.0. The mixed association mapping model used was Y = SNP*β + PC*α + K *µ + ε, where Y = response of the dependent variable (SDM percent), SNP = SNP marker (fixed effects), PC = principal component coordinate from the PCA (fixed effects), K = kinship matrix (random effects), α is the vector of PC, β and µ are the vectors of SNP and K, respectively, and ε is the error. In the linear models, the first 10 principal components were used as covariates. Manhattan plots were plotted using the −log 10 P values of all SNPs used in analysis; Q-Q plots were plotted of the observed −log 10 P values and the expected −log 10 P values to study the genomic inflation.

## Electronic supplementary material

Supplementary Dataset 1
