# Supplementary material for: Genome-wide association study in Asia-adapted tropical maize reveals novel and explored genomic regions for sorghum downy mildew resistance
Source: Sci Rep. 2018 Jan 10;8:366. doi: 10.1038/s41598-017-18690-3 (PMC5762920; doi:10.1038/s41598-017-18690-3)
Supplement: Supplementary file 1 — Supplementary Dataset 1 [file 41598_2017_18690_MOESM1_ESM.doc]

**Genome-wide association study in Asia-adapted tropical maize reveals novel and explored genomic regions for Sorghum downy mildew resistance.**

**Zerka Rashid^1^, Pradeep Kumar Singh^1^, Hindu Vemuri^1^, Pervez Haider Zaidi^1^, Boddupalli Maruthi Prasanna^2^ and Sudha Krishnan Nair^1^***

Supplementary Table 1. Chromosome-wise average adjacent pair distances between SNPs in which LD decayed at r^2^ = 0.2 and 0.1. The distances are given in Kb

|  |  |  | |
| --- | --- | --- | --- |
| **Chr.** | **r^2^ = 0.1** | **r^2^ = 0.2** |  |
| 1 | 19.09 | 6.62 |  |
| 2 | 11.39 | 3.95 |  |
| 3 | 14.32 | 4.97 |  |
| 4 | 22.14 | 7.68 |  |
| 5 | 10.35 | 3.59 |  |
| 6 | 13.26 | 4.6 |  |
| 7 | 14.51 | 5.03 |  |
| 8 | 28.8 | 9.99 |  |
| 9 | 18.12 | 6.29 |  |
| 10 | 16.31 | 5.66 |  |

Supplementary Table 2. Significant associations identified in GWAS corrected for population structure and kinship for SDM resistance in the CAAM panel

|  |  | |  | |  | |  | |  | |  | |  | |  | |
| --- | --- | --- | --- | --- | --- | --- | --- | --- | --- | --- | --- | --- | --- | --- | --- | --- |
| **Marker** | **Chr.** | **Position** | | **P-Value** | | **Bonferroni P** | | **Minor Allele Frequency** | | **Minor allele average** | | **Major allele average** | | **R^2^ (%)** | |  |
| S2_6154311 | 2 | 6154311 | | 3.48E-12 | | 0 | | 0.0493 | | 50.289 | | 81.19 | | 13.07 | |  |
| S3_51326624 | 3 | 51326624 | | 1.33E-10 | | 0 | | 0.0409 | | 58.256 | | 80.442 | | 11.257 | |  |
| S1_84567152 | 1 | 84567152 | | 1.37E-10 | | 0 | | 0.1531 | | 74.068 | | 81.748 | | 11.243 | |  |
| S1_84567165 | 1 | 84567165 | | 1.37E-10 | | 0 | | 0.1531 | | 74.068 | | 81.748 | | 11.243 | |  |
| S1_82633548 | 1 | 82633548 | | 3.49E-10 | | 0.0001 | | 0.1191 | | 72.577 | | 80.201 | | 10.771 | |  |
| S1_81579624 | 1 | 81579624 | | 1.12E-09 | | 0.0004 | | 0.1316 | | 73.554 | | 80.372 | | 10.181 | |  |
| S6_28477908 | 6 | 28477908 | | 1.65E-09 | | 0.0005 | | 0.1599 | | 73.373 | | 82.124 | | 9.987 | |  |
| S5_193651730 | 5 | 1.94E+08 | | 3.32E-09 | | 0.0011 | | 0.1535 | | 72.615 | | 80.666 | | 9.63 | |  |
| S6_145321547 | 6 | 1.45E+08 | | 8.66E-09 | | 0.0028 | | 0.0534 | | 63.207 | | 80.324 | | 9.141 | |  |
| S6_145310940 | 6 | 1.45E+08 | | 1.67E-08 | | 0.0055 | | 0.0446 | | 60.573 | | 80.287 | | 8.804 | |  |
| S6_145310942 | 6 | 1.45E+08 | | 1.67E-08 | | 0.0055 | | 0.0446 | | 60.573 | | 80.287 | | 8.804 | |  |
| S9_16183526 | 9 | 16183526 | | 1.72E-08 | | 0.0057 | | 0.0526 | | 63.511 | | 80.366 | | 8.789 | |  |
| S6_145491591 | 6 | 1.45E+08 | | 2.12E-08 | | 0.0069 | | 0.0652 | | 61.055 | | 80.561 | | 8.684 | |  |
| S6_145432240 | 6 | 1.45E+08 | | 2.89E-08 | | 0.0095 | | 0.0492 | | 63.207 | | 80.37 | | 8.524 | |  |
| S6_146125265 | 6 | 1.46E+08 | | 3.37E-08 | | 0.0111 | | 0.0735 | | 67.26 | | 80.425 | | 8.445 | |  |
| S6_145432233 | 6 | 1.45E+08 | | 4.13E-08 | | 0.0136 | | 0.0506 | | 63.207 | | 80.355 | | 8.34 | |  |
| S6_145312028 | 6 | 1.45E+08 | | 5.04E-08 | | 0.0166 | | 0.0653 | | 62.4 | | 80.373 | | 8.237 | |  |
| S8_173851044 | 8 | 1.74E+08 | | 5.68E-08 | | 0.0187 | | 0.0553 | | 55.08 | | 80.683 | | 8.176 | |  |
| S8_173851047 | 8 | 1.74E+08 | | 5.68E-08 | | 0.0187 | | 0.0553 | | 55.08 | | 80.683 | | 8.176 | |  |
| S8_173851049 | 8 | 1.74E+08 | | 5.68E-08 | | 0.0187 | | 0.0553 | | 55.08 | | 80.683 | | 8.176 | |  |
| S8_173851050 | 8 | 1.74E+08 | | 5.68E-08 | | 0.0187 | | 0.0553 | | 55.08 | | 80.683 | | 8.176 | |  |
| S8_173851053 | 8 | 1.74E+08 | | 5.68E-08 | | 0.0187 | | 0.0553 | | 55.08 | | 80.683 | | 8.176 | |  |
| S1_88996380 | 1 | 88996380 | | 8.67E-08 | | 0.0285 | | 0.1459 | | 70.893 | | 81.291 | | 7.958 | |  |
| S5_193536588 | 5 | 1.94E+08 | | 1.28E-07 | | 0.0421 | | 0.074 | | 66.917 | | 80.995 | | 7.756 | |  |
| S1_283192413 | 1 | 2.83E+08 | | 1.29E-07 | | 0.0424 | | 0.0461 | | 63.486 | | 79.939 | | 7.752 | |  |
| S3_28841309 | 3 | 28841309 | | 1.32E-07 | | 0.0434 | | 0.0507 | | 61.429 | | 80.223 | | 7.74 | |  |

Supplementary Table 3. Pair-wise LD between significantly associated SNPs for SDM resistance estimated as r^2^ values employing the Expectation Maximization (EM) method.

| **Chromosome** | **Marker 1** | **Marker 2** | **EM - r^2^** |
| --- | --- | --- | --- |
| 1 | S1_81579624 | S1_82633548 | 0.55 |
| 1 | S1_81579624 | S1_84567152 | 0.77 |
| 1 | S1_81579624 | S1_84567165 | 0.77 |
| 1 | S1_81579624 | S1_88996380 | 0.50 |
| 1 | S1_81579624 | S1_283192413 | 0.00 |
| 1 | S1_82633548 | S1_84567152 | 0.77 |
| 1 | S1_82633548 | S1_84567165 | 0.77 |
| 1 | S1_82633548 | S1_88996380 | 0.68 |
| 1 | S1_82633548 | S1_283192413 | 0.00 |
| 1 | S1_84567152 | S1_84567165 | 1.00 |
| 1 | S1_84567152 | S1_88996380 | 0.77 |
| 1 | S1_84567152 | S1_283192413 | 0.00 |
| 1 | S1_84567165 | S1_88996380 | 0.77 |
| 1 | S1_84567165 | S1_283192413 | 0.00 |
| 1 | S1_88996380 | S1_283192413 | 0.00 |
| 3 | S3_28841309 | S3_51326624 | 0.27 |
| 5 | S5_193536588 | S5_193651730 | 0.07 |
| 6 | S6_28477908 | S6_145310940 | 0.02 |
| 6 | S6_28477908 | S6_145310942 | 0.02 |
| 6 | S6_28477908 | S6_145312028 | 0.01 |
| 6 | S6_28477908 | S6_145321547 | 0.02 |
| 6 | S6_28477908 | S6_145432233 | 0.01 |
| 6 | S6_28477908 | S6_145432240 | 0.02 |
| 6 | S6_28477908 | S6_145491591 | 0.02 |
| 6 | S6_28477908 | S6_146125265 | 0.03 |
| 6 | S6_145310940 | S6_145310942 | 1.00 |
| 6 | S6_145310940 | S6_145312028 | 0.71 |
| 6 | S6_145310940 | S6_145321547 | 0.73 |
| 6 | S6_145310940 | S6_145432233 | 0.62 |
| 6 | S6_145310940 | S6_145432240 | 0.62 |
| 6 | S6_145310940 | S6_145491591 | 0.65 |
| 6 | S6_145310940 | S6_146125265 | 0.49 |
| 6 | S6_145310942 | S6_145312028 | 0.71 |
| 6 | S6_145310942 | S6_145321547 | 0.73 |
| 6 | S6_145310942 | S6_145432233 | 0.62 |
| 6 | S6_145310942 | S6_145432240 | 0.62 |
| 6 | S6_145310942 | S6_145491591 | 0.65 |
| 6 | S6_145310942 | S6_146125265 | 0.49 |
| 6 | S6_145312028 | S6_145321547 | 0.58 |
| 6 | S6_145312028 | S6_145432233 | 0.61 |
| 6 | S6_145312028 | S6_145432240 | 0.61 |
| 6 | S6_145312028 | S6_145491591 | 0.66 |
| 6 | S6_145312028 | S6_146125265 | 0.51 |
| 6 | S6_145321547 | S6_145432233 | 0.77 |
| 6 | S6_145321547 | S6_145432240 | 0.80 |
| 6 | S6_145321547 | S6_145491591 | 0.79 |
| 6 | S6_145321547 | S6_146125265 | 0.47 |
| 6 | S6_145432233 | S6_145432240 | 0.97 |
| 6 | S6_145432233 | S6_145491591 | 0.94 |
| 6 | S6_145432233 | S6_146125265 | 0.46 |
| 6 | S6_145432240 | S6_145491591 | 0.94 |
| 6 | S6_145432240 | S6_146125265 | 0.48 |
| 6 | S6_145491591 | S6_146125265 | 0.47 |
| 8 | S8_173851044 | S8_173851047 | 1.00 |
| 8 | S8_173851044 | S8_173851049 | 1.00 |
| 8 | S8_173851044 | S8_173851050 | 1.00 |
| 8 | S8_173851044 | S8_173851053 | 1.00 |
| 8 | S8_173851047 | S8_173851049 | 1.00 |
| 8 | S8_173851047 | S8_173851050 | 1.00 |
| 8 | S8_173851047 | S8_173851053 | 1.00 |
| 8 | S8_173851049 | S8_173851050 | 1.00 |
| 8 | S8_173851049 | S8_173851053 | 1.00 |
| 8 | S8_173851050 | S8_173851053 | 1.00 |

Supplementary Figure 1. The Scree plot of all the eigen values estimated in principal component analysis. The X-axis shows the components and the Y-axis shows the eigen values associated with each component.


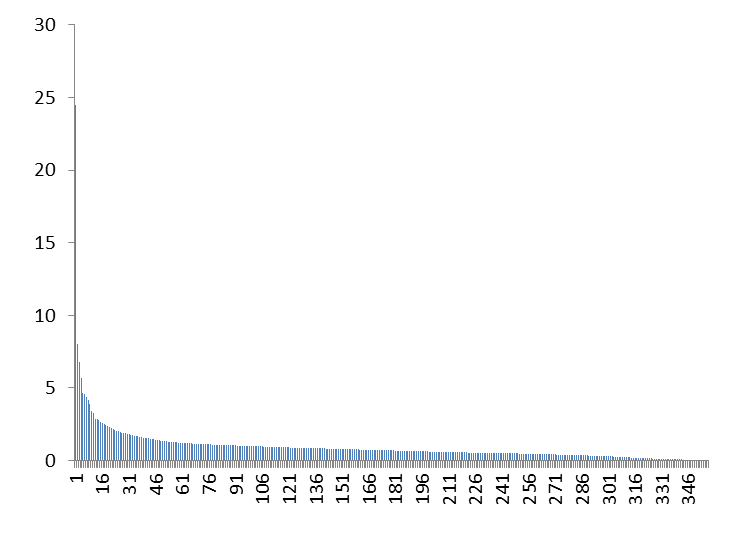


Components

Eigen Values
